# Krüppel-Like Factor 4 Is a Regulator of Proinflammatory Signaling in Fibroblast-Like Synoviocytes through Increased IL-6 Expression

**DOI:** 10.1155/2016/1062586

**Published:** 2016-06-16

**Authors:** Xinjing Luo, Jie Chen, Jianwei Ruan, Yongfeng Chen, Xuanrong Mo, Jiangwen Xie, Guoju Lv

**Affiliations:** ^1^Department of Basic Medical Sciences, School of Medicine, Taizhou University, Taizhou, Zhejiang 318000, China; ^2^Institute of Tumor Research, School of Medicine, Taizhou University, Taizhou 318000, China; ^3^Department of Orthopedics and Sports Medicine, Taizhou Municipal Hospital, Taizhou 318000, China; ^4^Department of Cardiology, Yingzhou District Second People's Hospital, Ningbo 315000, China

## Abstract

Human fibroblast-like synoviocytes play a vital role in joint synovial inflammation in rheumatoid arthritis (RA). Proinflammatory cytokines induce fibroblast-like synoviocyte activation and dysfunction. The inflammatory mediator Krüppel-like factor 4 is upregulated during inflammation and plays an important role in endothelial and macrophage activation during inflammation. However, the role of Krüppel-like factor 4 in fibroblast-like synoviocyte activation and RA inflammation remains to be defined. In this study, we identify the notion that Krüppel-like factor 4 is higher expressed in synovial tissues and fibroblast-like synoviocytes from RA patients than those from osteoarthritis patients.* In vitro*, the expression of Krüppel-like factor 4 in RA fibroblast-like synoviocytes is induced by proinflammatory cytokine tumor necrosis factor-*α*. Overexpression of Krüppel-like factor 4 in RA fibroblast-like synoviocytes robustly induced interleukin-6 production in the presence or absence of tumor necrosis factor-*α*. Conversely, knockdown of Krüppel-like factor 4 markedly attenuated interleukin-6 production in the presence or absence of tumor necrosis factor-*α*. Krüppel-like factor 4 not only can bind to and activate the interleukin-6 promoter, but also may interact directly with nuclear factor-kappa B. These results suggest that Krüppel-like factor 4 may act as a transcription factor mediating the activation of fibroblast-like synoviocytes in RA by inducing interleukin-6 expression in response to tumor necrosis factor-*α*.

## 1. Introduction

The autoimmune disease rheumatoid arthritis (RA) is characterized by persistent synovial inflammation and progressive joint destruction. Several cell types have been implicated in its pathogenesis, including lymphocytes, monocytes, and fibroblast-like synoviocytes (FLSs) [1–3]. FLSs, resident mesenchymal cells in the joint synovium, respond to proinflammatory stimuli including tumor necrosis factor-*α* (TNF-*α*) and interleukin-1 (IL-1) and exhibit features of inflammatory cells contributing to the pathogenesis of RA [1, 4]. Once activated, RA FLSs produce several types of cytokines and chemokines, including interleukin-6 (IL-6), interleukin-8 (IL-8), and macrophage inflammatory protein-1 (MIP-1) [5–7]. Of these proinflammatory mediators, IL-6 is an acute-phase inflammatory cytokine that plays a crucial role in joint inflammation and augments bone erosion in RA [8]. Blockade of IL-6 signal transduction represents a potentially useful therapeutic strategy to ameliorate RA inflammation [9, 10]. Given the importance of IL-6 in RA inflammation, identification of the molecular mechanisms regulating its expressions is of considerable importance. Induction of IL-6 by proinflammatory stimuli has been characterized and appears to occur mainly at the level of transcription. Several transcription factors have been implicated in this process [11]. The transcription factor nuclear factor-kappa B (NF-*κ*B) is activated in response to proinflammatory stimuli in RA FLSs and induces IL-6 gene expression [12].

Krüppel-like factors (KLFs) are a subclass of transcription factors that share homology with the* Drosophila* gene Krüppel [13]. These proteins contain zinc finger domains, located at the C-terminus, which bind to either CACCC elements or GC-boxes. The N terminus of these proteins mediates activation or repression of transcription and other protein-protein interactions [14]. Krüppel-like factor 4 (KLF4) is a member of the KLF family which was first found in the epithelial lining of the gut and skin and is involved in terminal differentiation and growth of epithelial cells [15, 16]. KLF4 has also been found to regulate stem cell function, cell survival, proliferation, and differentiation [17, 18]. KLF4 was recently implicated in the inflammation mediated by macrophages and endothelial cells [14, 19, 20] and is reported to be induced by several inflammatory stimuli and to play an important role in the production of inflammatory mediators. For example, in macrophages, KLF4 expression is upregulated in response to interferon-*γ* (IFN-*γ*), lipopolysaccharide (LPS), and TNF-*α* and interacts with the NF-*κ*B family member p65 to cooperatively activate the promoter of inducible nitric oxide synthase (iNOS) [14]. High-mobility group box 1 (HMGB1), an important late inflammatory cytokine during inflammation, participates in the pathogenesis of systemic inflammation and local inflammations, including sepsis and rheumatoid arthritis [21, 22]. It has been demonstrated that HMGB1 interacts with toll-like receptor-2 (TLR-2) and toll-like receptor-4 (TLR-4) to promote the activation of NF-*κ*B and mitogen-activated protein kinases (MAPK), leading to production of proinflammatory cytokines in macrophage and endothelial cells [23–25]. Recently, KLF4 binds to the promoter of HMGB1 and promotes its expression, translocation, and release in RAW 264.7 macrophages in response to LPS stimulation [26]. However, KLF4 may also perform anti-inflammatory functions. In human umbilical vein endothelial cells (HUVECs), KLF4 induced anti-inflammatory and antithrombotic factors, including endothelial nitric oxide synthase (eNOS) and thrombomodulin (TM), and inhibited expression of TNF-*α*-induced vascular cell adhesion molecule-1 (VCAM1) and tissue factor (TF) [19]. Therefore, the role of KLF4 in inflammation appears to be pleiotropic and its proinflammatory or anti-inflammatory functions are cell-type dependent. However, the role of KLF4 in FLS activation and RA inflammation remains to be defined.

In this study, we examined whether KLF4 is expressed in synovial tissue and in FLSs isolated from RA patients and whether the proinflammatory cytokine TNF-*α* can induce expression of KLF4. We also assessed the effect of KLF4 on expression of the proinflammatory cytokine IL-6 in RA FLSs and the mechanism by which KLF4 regulates IL-6 gene expression.

## 2. Methods

### 2.1. Tissue Preparation and Cell Culture

Human synovial tissue samples were obtained from RA patients and osteoarthritis (OA) patients at joint replacement surgery. OA patients were enrolled in this study as a control. All RA patients fulfilled the American College of Rheumatology 1987 criteria for RA [27]. The criteria are as follows: morning stiffness in and around joints lasting at least 1 hour before maximal improvement; soft tissue swelling of 3 or more joint areas; swelling of the proximal interphalangeal, metacarpophalangeal, or wrist joints; symmetric swelling; rheumatoid nodules; the presence of rheumatoid factor; and radiographic erosion and/or periarticular osteopenia in hand and/or wrist joints. Rheumatoid arthritis is defined by the presence of 4 or more criteria. We included RA patients with a disease duration of at least six months. Patients were allowed to use certain disease-modifying antirheumatic drugs (DMARD) and nonsteroidal anti-inflammatory drugs (NSAID). Patients who received immunosuppressive agents and biological agent such as TNF-*α* inhibitor were excluded. This study was approved by the Institutional Ethics Committee of Taizhou University, and informed consent was provided from all participants. FLSs were isolated as previously described [28] and cultured in Dulbecco's modified Eagle's medium (Life Technologies, Carlsbad, California, USA) containing 100 IU/mL penicillin and 100 *μ*g/mL streptomycin, supplemented with 10% fetal bovine serum (Life Technologies) at 37°C, in 5% CO_2_, and at 95% humidity. Cells at passages 4 to 8 were used in the following experiments.

### 2.2. Immunohistochemistry

Expression of KLF4 in human synovium was assessed by immunohistochemistry assay as previously described [29]. Synovial tissue obtained from RA patients was fixed in 4% paraformaldehyde in phosphate-buffered saline (PBS) for 24 h and then imbedded in paraffin and sliced into 5 *μ*m sections. The sections were deparaffinized in toluene and rehydrated in a gradient of alcohols. Antigen retrieval of synovium was achieved by incubation at 100°C for 5 min and peroxidase quenching by treatment with 3% hydrogen peroxide in methanol for 10 min. The sections were further blocked with 2% normal goat serum for 1 h and then incubated with rabbit anti-human KLF4 antibody (1 : 100, Abcam, Cambridge, MA, USA) at 4°C overnight. Antibody binding was detected by incubation with a peroxidase-conjugated goat anti-rabbit IgG (1 : 500, Santa Cruz, CA, USA) at room temperature (RT) for 1 h. Staining was developed using diaminobenzidine (DAB) reagent (Boster, Wuhan, China), and counterstaining was performed with hematoxylin.

### 2.3. Immunocytochemistry

Cells, cultured on chamber slides, were fixed in 4% formaldehyde for 30 min at room temperature (RT). Slides were blocked with 2% bovine serum albumin (BSA) for 1 h at RT and then incubated with rabbit anti-human KLF4 (1 : 200, Abcam) overnight at 4°C, followed by Cy3-conjugated goat anti-rabbit IgG (1 : 100, Santa Cruz) for 2 h. Nuclei were stained with Hoechst 33342, and staining was visualized using fluorescence microscopy (Olympus, Tokyo, Japan).

### 2.4. Generation of Expression Constructs

Expression plasmids for KLF4 and NF-*κ*B were generated by reverse-transcription PCR (RT-PCR) and cloned into pcDNA3.1. All the constructs were confirmed by sequencing (commercially by Invitrogen) (data not shown). Transfection of RA FLSs was performed by following the manufacturer's instructions (Lipofectamine*™* 2000, Invitrogen, Carlsbad, California, USA).

### 2.5. KLF4 Knockdown Experiments

Short interfering RNA (siRNA) against human KLF4 (sense: 5′-GCA GCU UCA CCU AUC CGA UTT-3′) and scrambled siRNA (ScRNA) (sense: 5′-UUC UCC GAA CGU GUC ACG UTT-3′) were designed and synthesized by GenePharma (Shanghai, China). 50 nM of KLF4 siRNA or scrambled siRNA was incubated with Lipofectamine RNAiMAX (Invitrogen) for 5 min at RT before addition to the FLSs. After 24 h or 48 h, transfected cells were harvested and KLF4 knockdown and target gene expression were assessed by PCR and western blotting.

### 2.6. Quantitative Real-Time PCR

Total RNA was isolated from cultured cells using TRIzol reagent (Invitrogen) according to the manufacturer's protocol. RNA (1 *μ*g) was reverse-transcribed using the RevertAid First Strand cDNA Synthesis Kit (Thermo Fisher Scientific, Waltham, MA, USA). Real-time PCR was performed as previously described [30]. The following primers were used: KLF4 primers, 5′-GCA ATA TAA GCA TAA AAG ATC ACC T-3′ (sense) and 5′-AAC CAA GAC TCA CCA AGC ACC-3′ (antisense); IL-6, 5′-CCT CCA GAA CAG ATT TGA GAG TAG T-3′ (sense) and 5′-GGG TCA GGG GTG GTT ATT GC-3′ (antisense); GAPDH, 5′-CGC TGA GTA CGT CGT GGA GTC-3′ (sense) and 5′-GCT GAT GAT CTT GAG GCT GTT GTC-3′ (antisense). Real-time fluorescence detection was performed on a ABI Prism 7700 sequence detection system (Applied Biosystems, Foster City, CA). Reactions were carried out by mixing 10 *μ*L of power SYBR green PCR master mix (Applied Biosystems), cDNA (100 ng), and forward and reverse primers (0.2 *μ*M each) in a final PCR reaction volume of 20 *μ*L. The amplification parameters were as follows: 95°C for 3 min (1 cycle), 95°C for 15 s, and 60°C for 60 s (40 cycles). The fluorescent signal was plotted versus cycle number, and the cycle threshold (Ct) was determined. Product specificity was confirmed by melting curve analysis. The slope of the standard curve was used to determine the amplification efficiency. Samples were analyzed in duplicate, using housekeeping gene glyceraldehyde-3-phosphate dehydrogenase (GAPDH) as an endogenous control, and the results were quantified using the comparative Ct method [31]. The relative quantity of each sample was normalized by subtracting the Ct of GAPDH to the target gene ([Δ]Ct = Ct of target gene − Ct of GAPDH). The relative quantity of target gene in a test sample was determined by comparing normalized target quantity in a test sample to normalized target quantity in the reference sample (−[Δ][Δ]Ct). Values were expressed using the following formula: 2^−[Δ][Δ]Ct^.

### 2.7. Western Blot

Cultured cells were lysed in radioimmune precipitation assay buffer [50 mM Tris-HCl (pH 7.4), 1% Triton X-100, 0.5% sodium deoxycholate, 150 mM sodium chloride, and 1% Nonidet P-40] supplemented with the complete protease inhibitor cocktail (Sigma) for 40 min at 4°C. The homogenate was centrifuged for 15 min, and then the supernatant was collected and the protein concentration of the fractions was estimated by a standard Bradford assay. 20–30 *μ*g of each protein sample was reduced and denatured by boiling in 2x sodium dodecyl sulfate (SDS) sample buffer [100 mM Tris-HCl (pH 6.8), 200 mM dithiothreitol (DTT), 4% SDS, 20% glycerol, and 0.1% bromophenol blue]. The proteins were separated on 10% sodium dodecyl sulfate-polyacrylamide gel (SDS-PAGE) and wet-transferred onto polyvinylidene fluoride (PVDF) membrane (Millipore, MA, USA) at 380 mA for 60 min. Membranes were blocked in blocking buffer (5% skim milk, 0.2% Tween-20 in Tris-buffered saline) for 4 h at RT and incubated with the primary antibody [rabbit anti-KLF4 polyclonal antibody (1 : 2000, Abcam); rabbit anti-NF-*κ*B-p65 polyclonal antibody (1 : 2000, Cell Signaling Technology, MA, USA); rabbit anti-tubulin polyclonal antibody (1 : 1000, Cell Signaling Technology); rabbit anti-histone 3 (H3) monoclonal antibody (1 : 1000, Cell Signaling Technology); rabbit anti-GAPDH monoclonal antibody (1 : 1000, Cell Signaling Technology)] overnight at 4°C with gentle agitation. After three washes of ten minutes each in 1x PBS-Tween-20 (1x PBST), the membranes were incubated with goat anti-rabbit horseradish peroxidase conjugate (1 : 5000, Santa Cruz, CA, USA) for 1 h at RT. The membranes were rinsed again in 1x PBST. Immunoreactive bands were developed by enhanced chemiluminescence reagent (Millipore) and bands of interest were scanned and quantified with Band Leader software (Shanghai, China).

### 2.8. Luciferase Assay and Reporter Construct

The IL-6 promoter construct was generated by PCR amplification of the IL-6 promoter from human genomic DNA using the following primers: 5′-CGC CTC GAG TGG ATG TAT GCT CCC GAC TT-3′ (forward) and 5′-CGC AAG CTT GCT ACA GAC ATC CCC AGT CTC-3′ (reverse). The fragment was cloned into the pGL3 vector, and the construct was confirmed by sequencing. For the luciferase reporter assay, RA FLSs were seeded in 24-well culture plates and 24 h later transfected using Lipofectamine 2000 transfection reagent according to the manufacturer's recommendations (Invitrogen). Each transfection was performed with 500 ng of pGL3-IL-6 promoter reporter construct, with or without transcription factor plasmid, and 20 ng of pRL-null control plasmid, in triplicate. Transfected cells were treated with TNF-*α* (Sigma) for the indicated time. After cell lysis, supernatants were collected and luciferase activity was detected using the Promega dual luciferase reporter assay system according to the manufacturer's instructions (Promega, Wisconsin, USA).

### 2.9. Electrophoretic Mobility Shift Assay (EMSA)

EMSA was performed using nuclear extract from RA FLSs using a LightShift Chemiluminescent EMSA Kit according to the manufacturer's instructions (Pierce Biotechnology, Rockford, USA). Biotin-labeled DNA probes for the KLF4 binding sites at positions −109 to −90 bp and −132 to −102 bp of the IL-6 promoter were generated as previously described [32]. Briefly, nuclear extract (5 *μ*g) was incubated with biotin-labeled DNA probes or unlabeled cold probe at RT for 20 min. The reaction mixtures were electrophoresed on 6% polyacrylamide gels and wet-transferred onto a nylon membrane (Millipore, MA, USA). After transfer, the protein-bound probe and free probe were immobilized using a UV cross-linker. The membranes were blocked and then incubated with streptavidin-horseradish peroxidase conjugate solution. After washing with wash buffer supplied by the manufacturer, the membrane was incubated in substrate equilibration buffer supplied by the manufacturer for 5 min, and immunoreactive bands were developed by enhanced chemiluminescence reagent (Millipore). For supershift assays, the nuclear extract was preincubated with rabbit anti-KLF4 antibody (Abcam) at 4°C for 1 h before biotin-labeled probes were added to the binding reaction mixture.

### 2.10. Coimmunoprecipitation (Co-IP)

Immunoprecipitation (IP) was performed using nuclear extract from RA FLSs as previously described [30]. Nuclear extracts (50 *μ*g) of RA FLSs were incubated with antibodies directed towards NF-*κ*B-p65 (1 *μ*g, Cell Signaling Technology), KLF4 (1 *μ*g, Abcam), and rabbit IgG (1 *μ*g) in IP buffer (50 mM Tris-HCl (pH 7.6), 150 mM sodium chloride, 1% Nonidet P-40, and 0.5% sodium deoxycholate) at 4°C overnight. 10 *μ*L of Sepharose G beads (GE Healthcare Bio-Sciences AB, Uppsala, Sweden) was rinsed with IP buffer [50 mM Tris-HCl (pH 7.6), 50 mM sodium chloride, 0.1% Nonidet P-40, and 0.05% sodium deoxycholate] and mixed with the antibody-incubated supernatant at RT for 4 h with gentle shaking. Then, the mixture was centrifuged at 4°C and 890 g for 3 min to collect the beads. The beads were washed in IP buffer (50 mM Tris-HCl (pH 7.6), 0.1% Nonidet P-40, and 0.05% sodium deoxycholate) and then mixed with 2x SDS loading buffer and boiled for 5 min. The supernatant was collected by centrifugation and loaded onto the gel for immunoblotting (as previously described).

### 2.11. Enzyme-Linked Immunosorbent Assay (ELISA)

RA FLSs were incubated with TNF-*α* (Sigma) for the indicated times. Supernatants were harvested and IL-6 concentrations were determined using sandwich ELISA, following the manufacturer's instructions (eBioscience). Absorbance at 450 nm was measured with a microplate reader (Bio-Rad, USA). A standard curve was generated by plotting absorbance versus log recombinant human IL-6 concentration. IL-6 was quantitated from a standard curve including known amounts of recombinant human IL-6. All data were normalized by cell number.

### 2.12. Statistical Analysis

Data was expressed as mean ± SEM. Unpaired Student's *t*-test was used for comparison between two groups. One-way analysis of variance (ANOVA) followed by Tukey-Kramer* post hoc* test was used for multiple comparisons. A *P* value of <0.05 was considered to represent statistical significance.

## 3. Results

### 3.1. KLF4 Expression in the Human Synovial Tissues of RA Patients

**Figure 1:**
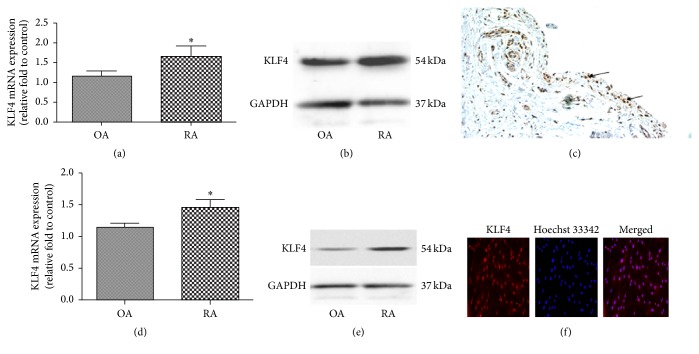
KLF4 expression in synovial tissue and FLSs from RA patients. Real-time PCR (a) and western blot (b) were performed to assess KLF4 expression in synovial tissues from patients with RA (*n* = 9) and OA (*n* = 4). GAPDH as an endogenous control. ^*∗*^ *P* < 0.05 in comparison to OA group. (c) KLF4 expression in synovial tissues from patients with RA was assessed by immunohistochemistry. Arrows indicate KLF4-positive cells (200x magnification). Real-time PCR (d) and western blot (e) were performed to assess KLF4 expression in isolated FLSs from patients with RA (*n* = 9) and OA (*n* = 4). GAPDH as an endogenous control. ^*∗*^ *P* < 0.05 in comparison to OA group. (f) KLF4 expression in isolated FLSs from patients with RA was assessed by immunofluorescence. Cell nuclei were visualized by Hoechst 33342 staining (100x magnification).

We used real-time PCR and western blotting to investigate whether KLF4 is expressed in the synovial tissues from patients with RA and OA. KLF4 expression was found in the synovial tissues from patients with both RA and OA, and KLF4 expression was higher in RA than in OA (Figures 1(a) and 1(b)). Immunohistochemistry revealed that KLF4-positive cells in the synovial tissues from RA patients were found predominantly in the synovial lining (Figure 1(c)).

To investigate whether this pattern of KLF4 expression is reflected in cultured primary FLSs isolated from the synovial tissues from patients with RA and OA, we harvested total mRNA and protein from these cells. RT-PCR and western blotting verified that KLF4 is expressed in cultured FLSs from patients with both RA and OA, and KLF4 expression was higher in RA than in OA (Figures 1(d) and 1(e)). Immunofluorescence revealed that KLF4 was localized in the nuclei of RA FLSs (Figure 1(f)).

### 3.2. TNF-*α* Induces KLF4 and IL-6 Gene Expression in RA FLSs

**Figure 2:**
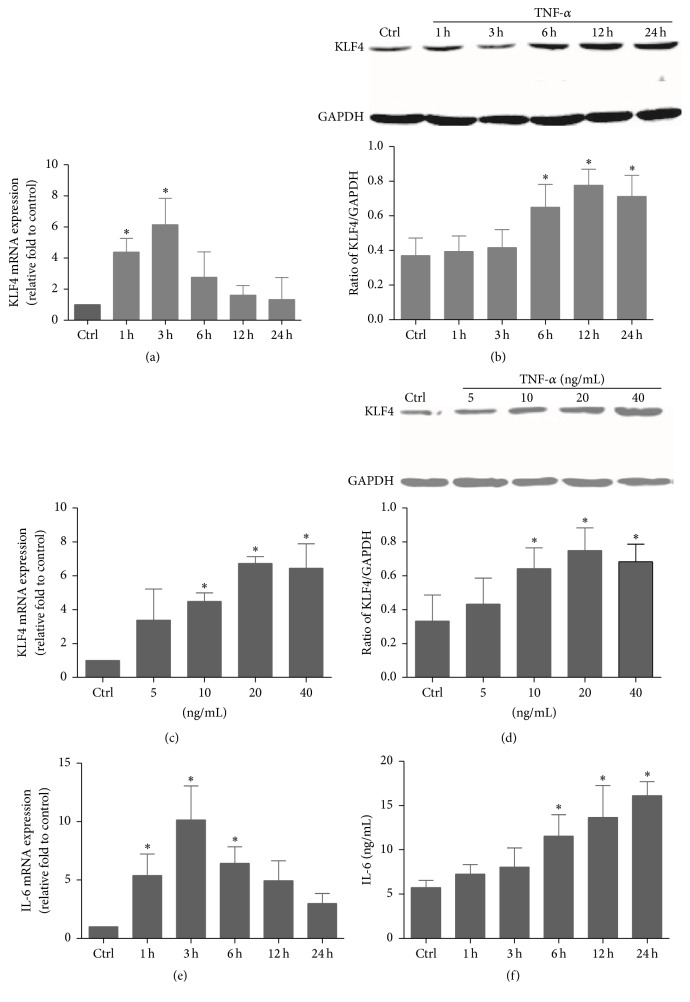
Expression of KLF4 and IL-6 in RA FLSs in response to TNF-*α*. KLF4 is induced by TNF-*α* in a time- and dose-dependent fashion. RA FLSs were incubated with 20 ng/mL TNF-*α* for the indicated time periods, and expression of KLF4 was assessed by real-time PCR (a) and western blotting (b). RA FLSs were incubated with TNF-*α* at different doses, and expression of KLF4 was assessed by real-time PCR after 3 h (c) and western blotting after 12 h (d). The lower panels in (b) and (d) indicate the ratio of KLF4/GAPDH protein. Time course of RA FLS IL-6 expression in response to 20 ng/mL TNF-*α* was assessed by real-time PCR (e) and ELISA (f). GAPDH as an endogenous control. Data were expressed as means ± SEM (*n* = 3); ^*∗*^ *P* < 0.05 in comparison to the untreated cells (Ctrl).

Previous studies have demonstrated that KLF4 is induced by proinflammatory stimuli in several cell types including macrophages and endothelial cells [14, 19]. However, the influence of proinflammatory stimuli on its expression in RA FLSs remains unclear. To determine whether KLF4 can be induced in human RA FLSs under inflammatory conditions, cells were treated with different doses of TNF-*α* for various periods of time. We found that the level of KLF4 mRNA and protein rapidly increased in RA FLSs incubated with TNF-*α* (20 ng/mL) within 24 h (Figures 2(a) and 2(b)). The expression of KLF4 mRNA and protein was peaked after 3 h and 16 h incubation with TNF-*α*, respectively. TNF-*α* elevated expression of both KLF4 mRNA and protein in a concentration-dependent manner. Maximal expression was observed in cells incubated with 20 to 40 ng/mL TNF-*α* (Figures 2(c) and 2(d)).

FLS activation and inflammation in RA are associated with upregulation of several proinflammatory cytokines. IL-6 is reported to be the key proinflammatory cytokine produced by FLSs in the RA synovium [33]. Thus, we analyzed the influence of TNF-*α* on IL-6 expression in FLSs. As shown in Figure 2(e), IL-6 mRNA levels in FLSs were significantly increased after incubation with TNF-*α* for 1 to 6 h and peaked at 3 h. Similarly, levels of IL-6 protein also increased significantly after incubation with TNF-*α* for 3 to 24 h and peaked at 24 h (Figure 2(f)). The increase in the level of IL-6 corresponded well to the increase in the level of KLF4 in RA FLSs induced by TNF-*α*, suggesting a relationship between these two proteins.

### 3.3. Overexpression of KLF4 Increases IL-6 Expression in RA FLSs

Because KLF4 is induced by TNF-*α* and TNF-*α*-induced KLF4 expression corresponded to TNF-*α*-induced IL-6 expression in RA FLSs, we considered the possibility that KLF4 may regulate IL-6 expression.

**Figure 3:**
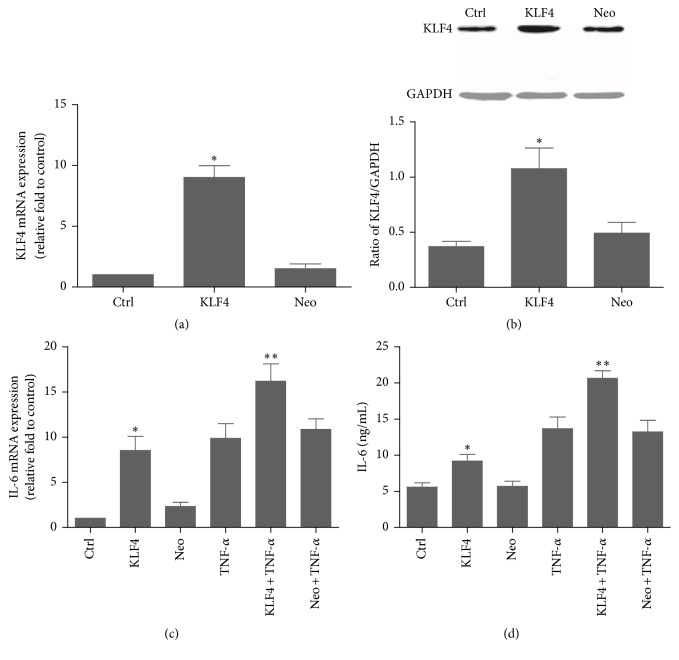
KLF4 overexpression induces IL-6 expression in RA FLSs. RA FLSs were transiently transfected with pcDNA3.1-KLF4 (KLF4) or the pcDNA3.1 empty vector (Neo) and then incubated with TNF-*α* (20 ng/mL). Expression of KLF4 was assessed after 3 h by real-time PCR (a) and after 12 h by western blotting (b). Expression of IL-6 was assessed after 3 h by real-time PCR (c) and ELISA (d). GAPDH as an endogenous control. Data were expressed as mean ± SEM (*n* = 3); ^*∗*^ *P* < 0.05 in comparison to the untreated cells (Ctrl); ^*∗∗*^ *P* < 0.05 in comparison to the TNF-*α* group.

In order to investigate the effect of KLF4 on expression of IL-6, RA FLSs were transfected with a pcDNA3.1-KLF4 construct. Overexpression of KLF4 induced significant increases in cellular content of both IL-6 mRNA and protein in the absence of TNF-*α* (Figure 3). This effect was potently enhanced after TNF-*α* stimulation. These data indicate that KLF4 overexpression enhances expression of IL-6 in RA FLSs.

### 3.4. Knockdown of KLF4 Decreases Expression of IL-6 in RA FLSs

**Figure 4:**
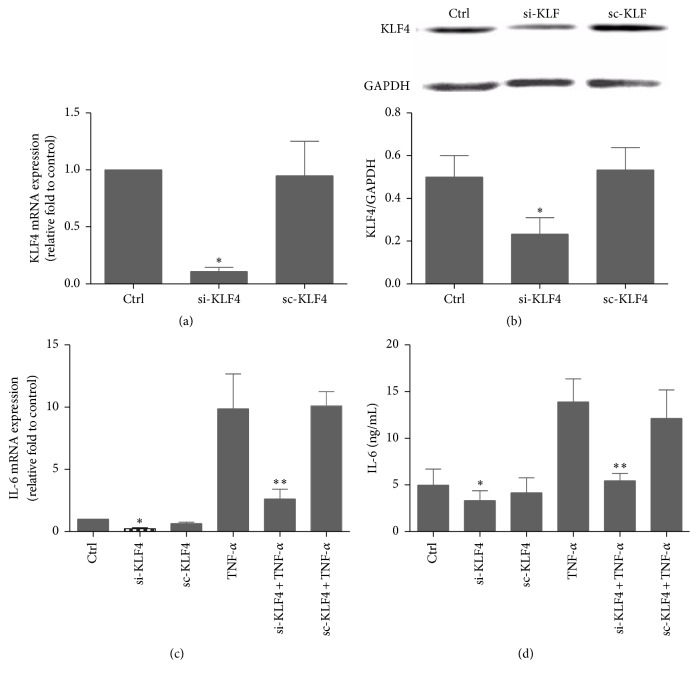
Effect of KLF4 knockdown on IL-6 expression in RA FLSs. RA FLSs were transiently transfected with KLF4 siRNA oligonucleotide (si-KLF4) or KLF4 scrambled siRNA (sc-KLF4) for 48 h and then incubated with TNF-*α* (20 ng/mL). KLF4 inhibition was assessed after 3 h by real-time PCR (a) and after 12 h by western blot (b). IL-6 expression was assessed by real-time PCR (c) and ELISA (d). GAPDH as an endogenous control. Data were expressed as means ± SEM (*n* = 3); ^*∗*^ *P* < 0.05 in comparison to the untreated cells (Ctrl); ^*∗∗*^ *P* < 0.05 in comparison to the TNF-*α* group.

To further assess the role of KLF4 in regulating IL-6 expression, siRNA was used to knock down KLF4 expression in RA FLSs. The level of IL-6 mRNA and protein was significantly lower in siRNA-transfected cells than in ScRNA-transfected cells, both in the presence and in the absence of TNF-*α* (*P* < 0.05; Figure 4). Taken together, these results suggest that KLF4 can regulate IL-6 expression in RA FLSs.

### 3.5. KLF4 Regulates IL-6 Promoter Activity in RA FLSs

**Figure 5:**
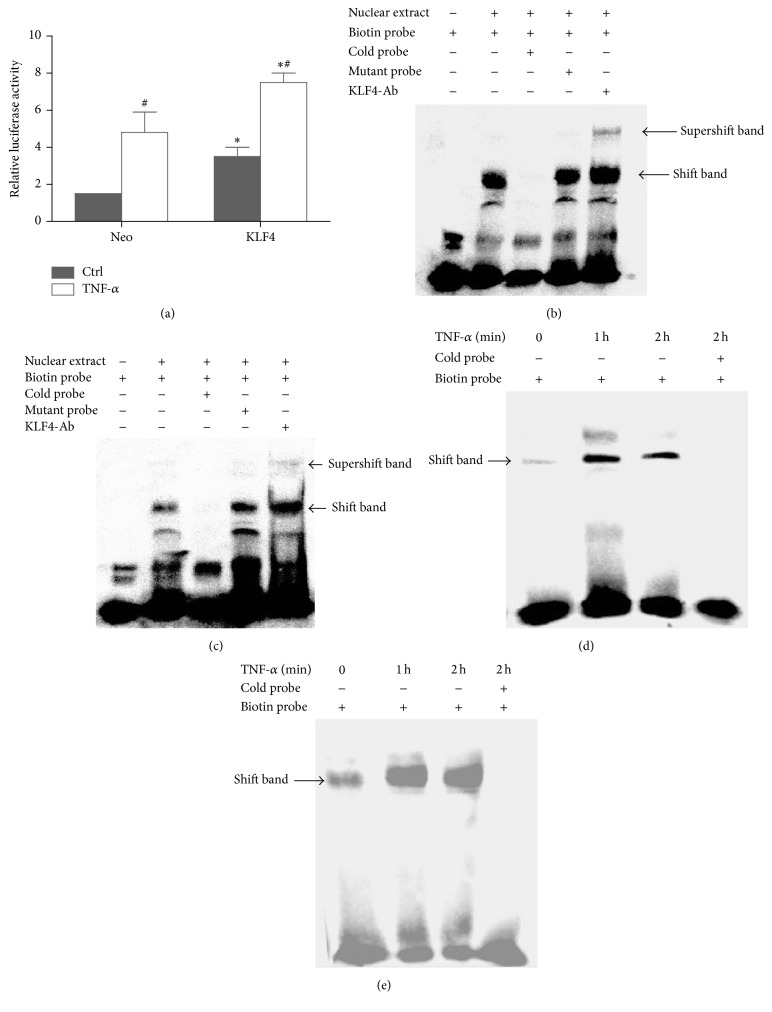
KLF4 regulates IL-6 promoter activity in RA FLSs. KLF4 transactivates the IL-6 promoter. RA FLSs were cotransfected in triplicate with the indicated IL-6 promoter luciferase plasmids, pcDNA3.1-KLF4 expression plasmid (KLF4), or pcDNA3.1 control vector (Neo) for 48 h and then incubated with TNF-*α* (20 ng/mL) for 12 h. Transcriptional activity was detected by the Dual Luciferase Assay (a). Data were expressed as means ± SEM (*n* = 3). ^*∗*^ *P* < 0.05 in comparison to the vector control group (Neo). ^#^ *P* < 0.05 in comparison to untreated group (Ctrl). KLF4 binds the IL-6 promoter. EMSA was performed with nuclear extracts using a biotinylated probe of the −109 to −90 bp site (b) and −132 to −102 bp site (below) in the IL-6 promoter (c). TNF-*α* augmented binding of KLF4 to the IL-6 promoter. EMSA was performed with nuclear extracts from cells incubated with TNF-*α* for 1 or 2 h. The previously described biotinylated probes (−109 to −90 bp and −132 to −102 bp) were used ((d) and (e), resp.).

To understand the mechanism by which KLF4 regulates IL-6 gene expression in RA FLSs, we investigated activation of the IL-6 promoter using a luciferase assay. As shown in Figure 5(a), KLF4 transactivated the IL-6 promoter in RA FLSs. IL-6 promoter activity was significantly higher in cells transfected with the KLF4 expression plasmid than in cells transfected with empty vector, both in the presence and in the absence of TNF-*α* stimulation (*P* < 0.05).

To determine whether KLF4 can bind the potential KLF4 binding element on the IL-6 promoter, EMSA experiments were performed using a biotinylated IL-6 probe for two segments of the IL-6 promoter (−109 to −90 bp and −132 to −102 bp), which are hypothesized to be important for KLF4 binding to the IL-6 promoter [32]. As shown in Figures 5(b) and 5(c), KLF4 binds to these two elements, and the addition of antibody against KLF4 led to a supershift of the band. The specificity of KLF4 for the probe sequence was confirmed using mutant oligonucleotides and cold probes. DNA-protein binding was significantly increased after TNF-*α* stimulation for 1 or 2 h (Figures 5(d) and 5(e)). Taken together, these results indicate that KLF4 interacts directly with the IL-6 promoter to regulate IL-6 expression.

### 3.6. KLF4 Potentially Interacts with NF-*κ*B to Induce the IL-6 Promoter

**Figure 6:**
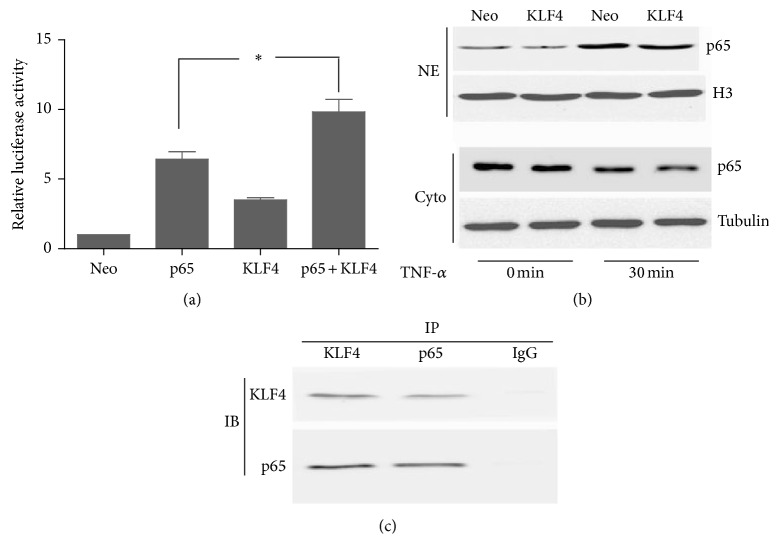
KLF4 and NF-*κ*B-p65 cooperatively induce the IL-6 promoter. KLF4 and NF-*κ*B-p65 cooperatively induce IL-6 promoter activity. RA FLSs were cotransfected with the indicated IL-6 promoter luciferase plasmids, pcDNA3.1-NF-*κ*B-p65 expression plasmid (p65), pcDNA3.1-KLF4 expression plasmid (KLF4), or the pcDNA3.1 empty vector (Neo). IL-6 promoter activity was measured by Dual Luciferase Assay (a). Data were expressed as means ± SEM (*n* = 3). ^*∗*^ *P* < 0.05 in comparison to the p65 group. KLF4 does not affect either expression or nuclear translocation of NF-*κ*B-p65. RA FLSs were transfected in triplicate with pcDNA3.1-KLF4 expression plasmid (KLF4) or pcDNA3.1 empty vector (Neo) and then incubated with TNF-*α* (20 ng/mL) for 30 min. Expression of NF-*κ*B-p65 (p65) in the cytoplasm and nucleus was detected by western blot. Cyto: cytoplasmic extracts; NE: nuclear extracts. Histone 3 (H3) and Tubulin served as controls for nuclear and cytoplasmic proteins. KLF4 associates with NF-*κ*B-p65. Nuclear extracts from RA FLSs were immunoprecipitated (IP) with anti-KLF4, anti-NF-*κ*B-p65 (p65), or anti-IgG antibodies, followed by immunoblotting (IB) with the indicated antibodies (c).

In response to inflammatory stimuli, the transcription factor NF-*κ*B has been demonstrated to induce IL-6 in RA FLSs [11]. The KLF4 binding sites on the IL-6 promoter are located in close proximity to the NF-*κ*B binding site. Therefore, we hypothesized that KLF4 and NF-*κ*B may interact directly. First, we investigated whether KLF4 could enhance induction of IL-6 expression by NF-*κ*B-p65. We found that KLF4 overexpression augmented the induction of the IL-6 promoter by NF-*κ*B (Figure 6(a)). Next, we assessed whether KLF4 mediates its effects* via* regulation of either NF-*κ*B expression or nuclear translocation. KLF4 overexpression did not increase expression of p65 or its nuclear accumulation in response to TNF-*α* (Figure 6(b)). Finally, we used coimmunoprecipitation studies to investigate whether KLF4 and NF-*κ*B directly interact. Nuclear extracts were probed with antibodies directed towards NF-*κ*B-p65 or KLF4, and we found that KLF4 was present in the complexes immunoprecipitated by the anti-NF-*κ*B-p65 antibody (Figure 6(c)). Conversely, NF-*κ*B-p65 was present in complexes precipitated by the anti-KLF4 antibody. Taken together, our findings suggest that KLF4 interacts physically with NF-*κ*B to cooperatively induce the IL-6 promoter.

## 4. Discussion

FLSs play a crucial role in joint inflammation and destruction in RA, mainly through production of proinflammatory cytokines. Several signaling pathways and transcription factors have been implicated in FLS activation and inflammation associated with RA [11, 34]. Recently, KLF4, a zinc finger-containing transcription factor, was reported to be involved in endothelial and macrophage-mediated inflammation [14, 19]. KLF4 was initially found in the epithelial lining of the gut and skin and was considered to be an epithelium-specific transcription factor that is involved in normal development of epithelia and carcinogenesis [35]. KLF4 was also recently reported to be expressed in the monocyte/macrophage lineage [14]. KLF4 regulates not only monocyte commitment and differentiation [36, 37], but also macrophage activation. KLF4 overexpression in J774a macrophages induced the macrophage activation marker iNOS and reduced expression of plasminogen activator inhibitor-1 [14]. Conversely, KLF4 knockdown inhibited iNOS induction by IFN-*γ* and/or LPS, whereas it led to enhanced responsiveness to TGF-*β*1 and Smad3 signaling [14]. In the RAW246.7 macrophage cell line, KLF4 has been shown to induce expression, translocation, and release of the late proinflammatory cytokine HMGB1 in response to LPS [26]. KLF4 expression has been demonstrated in microglial cells, the resident macrophages of the central nervous system, and it is significantly induced by LPS [30]. KLF4 knockdown in microglial cells reduced expression of TNF-*α*, MCP-1, IL-6, iNOS, and cyclooxygenase-2 (Cox-2) after induction by LPS [30]. Similar to mouse cells, KLF4 is markedly induced by proinflammatory cytokines including IFN-*γ*, LPS, or TNF-*α* and mediates proinflammatory signaling in human macrophages [14]. Several* in vitro* and* in vivo* studies [19, 20] demonstrated that human and mouse endothelial cells from arterial and venous blood vessels also expressed KLF4, and KLF4 was markedly induced by shear stress and inflammatory cytokines including TNF-*α*, IL-I*β*, and interferon-*γ*. KLF4 has been found to suppress inflammation by downregulating MCP-1, VCAM, and TM and upregulating eNOS and TF in endothelial cells [19]. Recent studies showed that KLF4 was highly expressed in thymocytes and mature T cells, and KLF4 directly binds to the promoter of IL-17 and positively regulates its expression [38]. The wide influence of KLF4 on inflammatory cells spurred our interest in exploring the relationship between KLF4 and RA. In this study, we established that KLF4 was expressed in synovial tissue and cultured FLSs isolated from both RA patients and OA patients, and KLF4 expression was higher in synovial tissue from RA patients than those from OA patients. We also found that the proinflammatory cytokine TNF-*α* induced KLF4 expression in RA FLSs in a time- and dose-dependent manner. Immunostaining revealed that KLF4 is mainly localized to the nucleus, in accordance with previous reports that found KLFs to be associated with specific regions of the nucleus, potentially with nucleoli [30, 39]. This data suggests that KLF4 is a transcription factor localized in nuclear regions of active transcription and regulates target gene expression in RA FLSs. Our results suggest a possible role for KLF4 in TNF-*α*-mediated inflammation in RA FLSs. While our studies have demonstrated that KLF4 expression was induced by TNF-*α* in RA FLSs in a dose-dependent manner, the relation between KLF4 expression in FLSs and TNF-*α* level* in vivo* has not been elucidated. Further investigations will also be needed to determine whether medications, including DMARGD or TNF inhibitors, affect KLF4 expression in RA FLSs.

Several cytokines and chemokines contribute to the progression and maintenance of inflammation in RA. Of these, FLS-derived IL-6 is considered to be crucially important and is therefore a target of RA drug development [12, 40]. IL-6 is a pleiotropic cytokine with a variety of biological activities including B- and T-cell activation, induction of autoantibody and peripheral blood platelet production, and release of acute-phase proteins [33]. In fact, increased levels of IL-6 have been found in the serum and synovial fluid of RA patients, and IL-6 levels were closely associated with disease activity [41–44]. IL-6 may both amplify the effects of TNF-*α* and IL-1 and stimulate the production of rheumatoid factors, acute-phase reactants, tissue-degrading enzymes, and other proinflammatory mediators [45, 46]. IL-6 has been well documented to play an indispensable role in RA inflammation, as blocking the IL-6 receptor can alleviate symptoms of RA [9, 10, 47]. In addition to macrophages and dendritic cells, human FLSs produce IL-6 and are considered to be a main source of IL-6 in the RA synovium [33]. IL-6 is spontaneously produced by cultured FLSs, and its expression is markedly upregulated by TNF-*α* [4]. Although TNF-*α* is a potent inducer of IL-6 production in human FLSs, the mechanism by which TNF-*α* induces IL-6 expression in FLSs remains unclear. Transcription factor NF-*κ*B was also reported to participate in regulation of IL-6 expression. Binding sites for NF-*κ*B have been identified in the promoter region of the IL-6 gene, and, under certain conditions, NF-*κ*B has been reported to activate transcription of the IL-6 gene and expression of IL-6 in RA FLSs [11, 12, 34].

Recently, the transcription factor KLF4 was reported to be essential for the regulation of inflammatory mediator gene expression in the primordial mesenchymal cell lineage, thus controlling generation of lymphocytes [18, 38], macrophages [14, 26, 35], and endothelial cells [19, 20]. Using bioinformatics analysis, we found that the IL-6 promoter contains KLF4 binding sites (CACCC). Importantly, we found that TNF-*α*-induced expression of KLF4 corresponded to IL-6 expression in RA FLSs. Therefore, we hypothesized that KLF4 might regulate IL-6 expression in RA FLSs. In this study, we demonstrated that KLF4 is essential for IL-6 production in RA FLSs. By experimentally overexpressing KLF4 or using KLF4 siRNA to downregulate KLF4 expression in RA FLSs, we found that expression of both IL-6 mRNA and protein was significantly upregulated after KLF4 overexpression and downregulated after KLF4 knockdown in the presence or absence of TNF-*α*. Our results indicate that KLF4 can promote IL-6 expression in RA FLSs, even under inflammatory conditions, suggesting that it may play a vital role in RA inflammation. Consistent with these proinflammatory effects, it has been reported that KLF4 can induce expression of IL-6 in microglia and dendritic cells [32]. However, in endothelial cells, KLF4 has been reported to suppress expression of IL-6 [19]. These observations indicate that the effect of KLF4 on the regulation of IL-6 expression varies with cell type; however, our findings indicate that, in FLSs, KLF4 regulates IL-6 expression, and this activity is likely relevant to the induction of IL-6 observed in RA.

KLF4 has previously been demonstrated to bind KLF4 DNA binding sites in the promoters of iNOS, TNF-*α*, IL-10, MCP-1, HMGB1, and IL-6 and to regulate expression of these inflammatory mediators [14, 26, 32, 35]. KLF4 has been reported to activate the iNOS promoter in macrophages by binding KLF4 binding sites at positions −95 and −212 bp [14]. KLF4 regulates HMGB1 expression in RAW264.7 macrophages by binding the KLF4 binding element in the HMGB1 promoter [26]. In dendritic cell lines, KLF4 activates expression of IL-6 primarily through binding to a proximal CACCC site in the IL-6 promoter [32]. In this study, we also demonstrated that KLF4 regulates transcription of IL-6 in RA FLSs by binding to two KLF4 binding elements in regions −109 to approximately −90 bp and −132 to approximately −102 bp from the transcriptional start site of the IL-6 promoter, and binding activity was enhanced after TNF-*α* stimulation. These results suggest that KLF4 regulates expression of IL-6 in RA FLSs by directly binding the IL-6 promoter.

The role of NF-*κ*B in the regulation of TNF-*α*-induced IL-6 expression in FLSs is well established. Interestingly, the KLF4 binding sites in the IL-6 promoter are close to NF-*κ*B binding site [32]. Kaushik et al. previously demonstrated that KLF4 interacts with pNF-*κ*B to cooperatively induce transcription of the iNOS and Cox-2 genes in microglial cells [30]. We hypothesized that KLF4 might exert its effects through direct interaction with other proteins such as NF-*κ*B in RA FLSs. Our results demonstrate that KLF4 interacts directly with NF-*κ*B to induce the IL-6 promoter in RA FLSs. We also considered the possibility that KLF4 exerts its effects through promoting expression or nuclear translocation of NF-*κ*B. However, we found that KLF4 overexpression did not affect either the expression or the nuclear accumulation of NF-*κ*B in either the presence or the absence of TNF-*α*. In addition, we did not find evidence that KLF4 expression and KLF4-NF-*κ*B binding in RA FLSs were altered by increased NF-*κ*B expression (data not shown). Therefore, our data suggest that KLF4 is a binding partner of NF-*κ*B and coregulates IL-6 expression in FLSs from RA patients. Of interest, contrary to our studies, KLF4 inhibited TNF-*α*-induced expression of the vascular cell adhesion molecule-1 (VCAM1) through blocking the binding of NF-*κ*B to VCAM1 promoter in cultured endothelial cells [48]. These differences may lie in the different types of cells used for the experiments. In addition, whether interaction of KLF4 with additional factors influences the regulation of IL-6 expression in RA FLSs needs further investigation. It has been shown that the interaction of HMGB1 and TLR4 leads to the activation of NF-*κ*B [23, 24]. It is possible that the HMGB1-TLR4 pathway induces KLF4 expression, which may subsequently result in enhanced activation of NF-*κ*B in FLSs.

## 5. Conclusions

In summary, we established KLF4 to be a TNF-*α*-induced transcription factor that regulates expression of the key proinflammatory cytokine IL-6 in RA FLSs through both direct promoter activation and interaction with NF-*κ*B. These results indicate that KLF4, which is induced by proinflammatory stimuli, can regulate FLSs activation and RA-associated inflammation. Studies on other target inflammatory mediator genes regulated by KLF4 are currently underway.
